# Self-administered Food Frequency Questionnaire Used in the 5-year Follow-up Survey of the JPHC Study: Questionnaire Structure, Computation Algorithms, and Area-based Mean Intake

**DOI:** 10.2188/jea.13.1sup_13

**Published:** 2007-11-30

**Authors:** Satoshi Sasaki, Minatsu Kobayashi, Junko Ishihara, Shoichiro Tsugane

**Affiliations:** 1 Epidemiology and Biostatistics Division, National Cancer Center Research Institute East.; 2 Cancer Information and Epidemiology Division, National Cancer Center Research Institute.

**Keywords:** food frequency questionnaire, nutrient, food group, diet

## Abstract

In this section we described the structure of the self-administered semiquantitative food frequency questionnaire used in the 5-year follow-up survey of the JPHC study, the computation algorithms, and the area-based mean intakes of nutrients and food groups in the subjects of the validation study. The FFQ consists of five sections: 1) semiquantitative frequency questions for rice and miso (fermented soybean paste)-soup, 2) those for alcoholic beverages, 3) those for vitamin supplements, 4) those for foods and beverages, and 5) questions on dietary and cooking behaviors. From the questions, intakes of nutrients and foods by food groups were computed. Although most of them were computed from the frequency and relative portion size indicated in the replies, together with the fixed portion size, a seasonal coefficient was added in the computation of vegetables and fruits. Only frequency of intake and fixed portion size were used for computation of beverages. Sugar and cream added in coffee and tea were computed from the frequency of coffee and tea intake. The intakes of cooking oil, cooking salt (sodium), and salt in noodle-soup were estimated from the questions of relative preference of oil, salt, and noodle-soup.

Self-administered semiquantitative food frequency questionnaires (FFQ) have been used to assess nutrient and food intake levels of the subjects in large-scale epidemiologic studies from several countries.^1^ The development of a FFQ most suited to the study objectives and subject characteristics is the key to obtaining fruitful results in a cohort study when this type of questionnaire is used for the assessment of dietary habits. The JPHC study group has developed a FFQ based on a data-based approach using 3-day dietary record data obtained from 335 subjects sampled from the 4 areas in the JPHC study, Cohort I,^2^ In this section, the questionnaire design of the FFQ and the computation algorithm are briefly presented together with the intake levels of main nutrients and food groups among the subjects of the validation study.

## MATERIALS AND METHODS

### Questionnaire Design and Computation Algorithm

For computation of food and nutrient intake from the FFQ, we divided the 138 food items and 14 supplementary questions reported in the previous publication about FFQ development 2 into five sections: 1) semiquantitative frequency questions regarding rice and miso (fermented soybean paste)-soup, 2) alcoholic beverages, 3) supplements, 4) 135 foods, and 5) 13 questions regarding dietary and cooking behaviors.

In the first section, questions on the relative size of the rice-bowl were asked in terms of 3 categories; small, medium-size, and large. The intake of rice was asked in terms of the number of bowls consumed, from less than 1, to 1, 2, 3, 4, 5, 6, 7-9, or 10 per day. Two additional questions about rice were asked: “Do you eat vitamin-enriched rice?”, and “Do you mix in other grains such as barley, foxtail millet, or Japanese barnyard millet?”. For miso-soup, the frequency of the intake was asked from almost never to 1-3 times/month, 1-2 times/week, 3-4 times/week, 5-6 times/week, or daily. The intake amount was asked in terms of the number of bowls from less than 1, to 1, 2, 3, 4, 5, 6, 7-9, to 10 per day. The relative salt content was also asked in terms of less salty, medium-salty, and salty.

In the second section, frequency of alcohol drinking was asked using 6 categories from almost never to 1-3 times/month, 1-2 times/week, 3-4 times/week, 5-6 times/week, or daily. The usual intake amount was asked using the standard units (volumes) for each type of alcoholic beverage from 1, 2, 3, 4, 5-6, to 7 units per occasion. The standard units in Japan are the following: go (used as a Japanese standard unit of alcoholic beverage, equivalent to ethanol in 180 ml of sake) for sake, shochu (distilled alcoholic beverage made of sweet potatoes, barley, rice, or other in Japan) and awamori (distilled alcoholic beverage made from rice in Okinawa); a large bottle of beer (633 ml); 1 glass (30 ml) for whisky; and 1 glass (60 ml) for wine.

In the third section, the use of vitamin supplements was asked in terms of the frequency and duration of use, and separately for supplements with multi-vitamins, including beta-carotene, vitamin C, and vitamin E. The frequency was from 1-2 pills/week, 3-4 pills/week, 5-6 pills/week, 1 pill/day, 2-3 pills/day, to 7 pills/day. The duration of use was from < 1 year, 1-2 years, 3-4 years, 5-9 years, 10-19 years, to > 20 years.

In the fourth section, the frequencies and relative portions or standard units were asked for 113 foods, and only frequency was asked for the 18 beverages. The food names and the standard portions/units were shown in Table 1. The frequency for the 113 foods was from never, 1-3 times/month, 1-2 times/week, 3-4 times/week, 5-6 times/week, once/day, 2-3 times/day, 4-6 times/day, to > 7 times/day. The beverage intake frequency was from 1-2 times/week, 3-4 times/week, 5-6 times/week, to one cup/day, 2-3 cups/day, 4-6 cups/day, 7-9 cups/day, or > 10 cups/day. The standard portions/units for each food were stated for 108 foods, and both in writing and in photo form for 5 vegetables. The relative portions/units used were from small (50% smaller) to medium and large (50% larger). Questions on the amount of sugar and milk used for tea and coffee were asked by 5 categories; none, half a spoon, one spoonful, two spoonfuls, and 3 spoonfuls for tea and coffee separately.

**Table 1.** Food items estimated from their intake from FFQ, their standard portion sizes, seasonal coefficients

	Food/Beverage Name	Standard portion size (g)	Semi-quantification^1^	Food group^2^	Seasonal coefficient^3^	Food used for development of food composition table					

						Food code^4^			Weighting ratio (%)		
1	Well-milled rice	140	yes	1	---	1-41d	Water	---	43	57	---
2	Vitamin supplemented rice	140	yes	1	---	1-41f	1-41d	Water	0.2	43.3	56.5
3	Rice mixed with other grains	140	yes	1	---	1-6a	1-41d	Water	7	36	57
4	Miso-soup	150	no	7	---	7-32c	Water	---	8	92	---
5	Sake	180	no	16	---	16-1b	---	---	100	---	---
6	Shochu, awamori	180	no	16	---	16-4c	---	---	100	---	---
7	Beer	633	no	16	---	16-2a	---	---	100	---	---
8	Whisky	80	no	16	---	16-5b	---	---	100	---	---
9	Wine	60	no	16	---	16-3a	16-3b	---	50	50	---
10	Steaks	150	yes	9	---	9-11b	---	---	100	---	---
11	Grilled beef	100	yes	9	---	9-7b	---	---	100	---	---
12	Stewed beef	50	yes	9	---	9-15b	---	---	100	---	---
13	Stir-fried pork	60	yes	9	---	9-69a	---	---	100	---	---
14	Deep-fried pork	100	yes	9	---	9-67a	---	---	100	---	---
15	Stewed pork, Western style	50	yes	9	---	9-71a	---	---	100	---	---
16	Stewed pork, Japanese style	60	yes	9	---	9-69a	---	---	100	---	---
17	Pork in soup	40	yes	9	---	9-69a	---	---	100	---	---
18	Pork liver	40	yes	9	---	9-80	---	---	100	---	---
19	Grilled chicken	70	yes	9	---	9-49b	---	---	100	---	---
20	Deep-fried chicken	50	yes	9	---	9-47b	---	---	100	---	---
21	Chicken liver	30	yes	9	---	9-55	---	---	100	---	---
22	Ham, loin	15	yes	9	---	9-86c	---	---	100	---	---
23	Sausage, Wieners	30	yes	9	---	9-87e	---	---	100	---	---
24	Bacon	20	yes	9	---	9-85a	---	---	100	---	---
25	Luncheon Meat	40	yes	9	---	9-31	---	---	100	---	---
26	Milk	200	yes	11	---	11-2	---	---	100	---	---
27	Egg	50	yes	10	---	10-5a	---	---	100	---	---
28	Cheese	20	yes	11	---	11-23	---	---	100	---	---
29	Yogurt	120	yes	11	---	11-9a	---	---	100	---	---
30	Salted fish	70	yes	8	---	8-114	8-146a	8-79	30	20	50
31	Dried fish	50	yes	8	---	8-5a	---	---	100	---	---
32	Canned tuna	20	yes	8	---	8-153c	---	---	100	---	---
33	Salmon, trout	70	yes	8	---	8-77	8-154	---	80	20	---
34	Bonito, tuna	60	yes	8	---	8-50	8-150a	---	50	50	---
35	Cod, flat fish	40	yes	8	---	8-113	8-60a	---	60	40	---
36	Sea bream	70	yes	8	---	8-110a	---	---	100	---	---
37	Horse mackerel, sardine	80	yes	8	---	8-4a	8-26a	---	50	50	---
38	Pacific sawry, mackerel	80	yes	8	---	8-95a	8-84a	---	50	50	---
39	Dried small fish	10	yes	8	---	8-32	---	---	100	---	---
40	Roe	20	yes	8	---	8-118a	8-81	---	60	40	---
41	Eel	50	yes	8	---	8-41	---	---	100	---	---
42	Squid	50	yes	8	---	8-206a	---	---	100	---	---
43	Octopus	50	yes	8	---	8-238b	---	---	100	---	---
44	Prawn	40	yes	8	---	8-219a	---	---	100	---	---
45	Short-necked clam, crab shell	20	yes	8	---	8-171	---	---	100	---	---
46	Vivipara	20	yes	8	---	8-185	---	---	100	---	---
47	Chikuwa (fish paste product)	20	yes	8	---	8-250	---	---	100	---	---
48	Kamaboko (fish paste product)	20	yes	8	---	8-246	---	---	100	---	---
49	Carrot	50	yes	12g	0.73	12-94a	---	---	100	---	---
50	Spinach	50	yes	12g	0.62	12-117a	---	---	100	---	---
51	Pumpkin	40	yes	12g	0.64	12-17a	---	---	100	---	---
52	Cabbage	30	yes	12	0.92	12-24a	---	---	100	---	---
53	Chinese radish	80	yes	12	0.53	12-56a	---	---	100	---	---
54	Salted pickles of Chinese radish	30	yes	12p	0.56	12-58	---	---	100	---	---
55	Salted pickles of green leafy vegetables	30	yes	12p,g	0.58	12-98b	---	---	100	---	---
56	Pickled plum	68	yes	13,12p	0.90	13-14	---	---	100	---	---
57	Pickled Chinese cabbage	30	yes	12p	0.54	12-101b	---	---	100	---	---
58	Pickled cucumber	30	yes	12p	0.36	12-25b	---	---	100	---	---
59	Pickled egg plant	30	yes	12p	0.34	12-87c	---	---	100	---	---
60	Sweet pepper	30	yes	12g	0.44	12-108a	---	---	100	---	---
61	Tomato	50	yes	12	0.39	12-85	---	---	100	---	---
62	Chinese chive	20	yes	12	0.50	12-93a	---	---	100	---	---
63	Garland chrysanthemum	30	yes	12g	0.68	12-39a	---	---	100	---	---
64	Komatsuna	20	yes	12g	0.63	12-32a	---	---	100	---	---
65	Broccoli	30	yes	12g	0.70	12-114a	---	---	100	---	---
66	Onion	50	yes	12	0.95	12-70a	---	---	100	---	---
67	Cucumber	30	yes	12	0.53	12-25a	---	---	100	---	---
68	Chinese cabbage	30	yes	12	0.58	12-101a	---	---	100	---	---
69	Bean sprout	25	yes	12	0.76	12-128a	---	---	100	---	---
70	Snap bean	30	yes	12g	0.36	12-6a	---	---	100	---	---
71	Lettuce	10	yes	12	0.44	12-73	---	---	100	---	---
72	Chingensai	70	yes	12g	0.50	12-74a	---	---	100	---	---
73	Leaf mustard	70	yes	12g	0.52	12-19a	---	---	100	---	---
74	Bitter gourd	100	yes	12	0.50	12-92a	---	---	100	---	---
75	Chard, Swiss chard	100	yes	12g	0.50	12-113a	---	---	100	---	---
76	Loofah	100	yes	12	0.50	12-115a	---	---	100	---	---
77	Mugwort	10	yes	12g	0.32	12-134a	---	---	100	---	---
78	Papaya	50	yes	13	0.90	13-66a	---	---	100	---	---
79	Mandarin orange	14	yes	13	0.51	13-17b	---	---	100	---	---
80	Other oranges	75	yes	13	0.49	13-62	13-24b	13-11	30	60	10
81	Apple	85	yes	13	0.61	13-88	---	---	100	---	---
82	Persimmon	80	yes	13	0.20	13-26a	---	---	100	---	---
83	Strawberries	75	yes	13	0.48	13-6	---	---	100	---	---
84	Grapes	100	yes	13	0.30	13-70	---	---	100	---	---
85	Melon	60	yes	13	0.36	13-80a	---	---	100	---	---
86	Watermelon	120	yes	13	0.27	13-45	---	---	100	---	---
87	Peach	65	yes	13	0.25	13-81a	---	---	100	---	---
88	Pear	80	yes	13	0.35	13-52a	---	---	100	---	---
89	Kiwifruit	50	yes	13	0.47	13-31	---	---	100	---	---
90	Pineapple	130	yes	13	0.61	13-58	---	---	100	---	---
91	Banana	75	yes	13	0.61	13-64	---	---	100	---	---
92	Breads	60	yes	1	---	1-13a	---	---	100	---	---
93	Udon	250	yes	1	---	1-23a	Water	---	26	74	---
94	Soba	200	yes	1	---	1-62a	Water	---	26	74	---
95	Okinawa soba	200	yes	1	---	1-30a	Water	---	26	74	---
96	Chinese noodles	220	yes	l	---	1-28a	Water	---	26	74	---
97	Mochi	50	yes	1	---	1-47	---	---	100	---	---
98	Japanese-style confectionery	70	yes	4	---	4-18	4-24e	---	30	70	---
99	Cake	70	yes	4	---	4-46	---	---	100	---	---
100	Biscuit, cookie	25	yes	4	---	4-65a	4-65c	---	30	70	---
101	Chocolate	25	yes	4	---	4-77a	---	---	100	---	---
102	Peanuts	20	yes	6	---	6-25b	---	---	100	---	---
103	Tofu for miso-soup	20	yes	7	---	7-21a	---	---	100	---	---
104	Tofu for other dishes	75	yes	7	---	7-21a	---	---	100	---	---
105	Yushi-dofu	150	yes	7	---	7-22	Water	---	47	53	---
106	Freeze-dried tofu	60	yes	7	---	7-27	---	---	100	---	---
107	Deep-fried tofu	72	yes	7	---	7-25	---	---	100	---	---
108	Natto	50	yes	7	---	7-29	---	---	100	---	---
109	Sweet potato	40	yes	2	---	2-5a	---	---	100	---	---
110	Potato	50	yes	2	---	2-11a	---	---	100	---	---
111	Yam	30	yes	2	---	2-8a	---	---	100	---	---
112	Shiitake mushroom	20	yes	14	---	14-7a	Water	---	20	80	---
113	Enokitake, Shimeji	20	yes	14	---	14-1a	14-10a	---	50	50	---
114	Wakame	20	yes	15	---	15-35a	Water	---	13	88	---
115	Hijiki	20	yes	15	---	15-28	Water	---	20	80	---
116	Nori	62	yes	15	---	15-4	15-5	---	70	30	---
117	Butter for spread	78	yes	5	---	11-28	---	---	100	---	---
118	Margarine for spread	88	yes	5	---	5-7a	---	---	100	---	---
119	Salad dressing	10	yes	17	---	17-9a	---	---	100	---	---
120	Mayonnaise	7	yes	17	---	17-10a	---	---	100	---	---
121	Worchester sauce	15	yes	17	---	17-5a	---	---	100	---	---
122	Ketchup	25	yes	17	---	17-6a	---	---	100	---	---
123	Soy milk	200	no	7	---	7-39b	---	---	100	---	---
124	Green tea (sencha)	120	no	16	---	16-21b	---	---	100	---	---
125	Green tea (bancha, genmaicha)	120	no	16	---	16-23b	16-25b	---	70	30	---
126	Oolong tea	120	no	16	---	16-26b	---	---	100	---	---
127	Tea	120	no	16	---	16-27b	---	---	100	---	---
128	Coffee (not canned coffee)	120	no	16	---	16-30b	---	---	100	---	---
129	Canned coffee	120	no	16	---	16-31	---	---	100	---	---
130	Soup	16	no	16	---	18-4	Water	---	22	78	---
131	Lactic acid bacteria beverage	65	no	16	---	11-10a	---	---	100	---	---
132	100% orange juice	200	no	16	---	13-19a	---	---	100	---	---
133	100% apple juice	200	no	16	---	13-89a	---	---	100	---	---
134	Tomato juice	200	no	12	---	12-86b	---	---	100	---	---
135	Calcium fortified soft drink	200	no	---	---	---	---	---	---	---	---
136	Beta-carotene fortified soft drink	200	no	---	---	---	---	---	---	---	---
137	Soft drink	250	no	16	---	16-33b	---	---	100	---	---
138	Vitamin-fortified soft drink)	200	no	---	---	---	---	---	---	---	---
139	Tap or well water	200	no	---	---	Water	---	---	100	---	---
140	Mineral water and filtered water	200	no	---	---	Water	---	---	100	---	---
141	Sugar for tea	3	no	---	---	3-4a	---	---	100	---	---
142	Sugar for coffee	5	no	---	---	11-8	---	---	100	---	---
143	Cream for tea	3	no	---	---	3-4a	---	---	100	---	---
144	Cream for coffee	5	no	---	---	11-8	---	---	100	---	---
145	Cooking oil	no 5	---	5	---	5-17	---	---	100	---	---
146	Cooking salt (sodium)	no 5	---	17	---	17-2a	---	---	100	---	---
147	Salt (sodium) in noodle-soup	no 5	---	---	---	17-2a	Water	---	1	99	---

^1^The food items with portion sizes were indicated as "yes".

^2^The definition of food group was based on the Standard Tables of Food Composition in Japan, the fourth revised edition. g=green and yellow vegetable, p=salted and pickled vegetable.

^3^Seasonal coefficient used to estimate the average yearly intake year for each food.

^4^Food code in the Standard Tables of Food Composition in Japan, the fourth revised edition.

^5^Estimated from combination of food intakes and answers to dietary behavior questions.

In the fifth section on dietary and cooking behaviors, 13 questions were asked. These questions are described elsewhere in this supplement.^2^

### Computation of Food and Nutrient Intakes

Intakes of rice and miso from miso-soup were calculated from bowl size, frequency, and the number of bowls consumed per day. Standard bowl sizes were 140 grams for rice and 150 grams for miso-soup for both sexes. The portion of rice for a small bowl and a large bowl was 110 and 170 grams, respectively. In the category of rice, rice boiled with cooking water was used for food and nutrient computations, while for miso-soup, miso and cooking water were used. Cooking water was not considered in other food computations.

For alcohol (ethanol), the following ethanol content was used for the calculation: 180 ml sake, 23 grams of ethanol; 180 ml shochu and awamori, 36 grams of ethanol; 30 ml whisky or brandy, 10 grams of ethanol; 60 ml wine, 6 grams of ethanol; and 633 ml beer, 6 grams of ethanol.

For the computation of the total dietary intake covering all but the third section dealing with the use of vitamin supplements, we developed a composition table for 147 foods and beverages from food items and supplemental questions on the FFQ (Table 1). The composition values of 147 foods were multiplied by the frequencies and the relative portion sizes for the food items from the FFQ. For 18 beverages without unit volume questions, the standard unit was used for the computation. The coefficients for the categories of relative portion sizes were 0.5 for small, 1.0 for medium, and 1.5 for large. For frequencies, the median frequency was used, for example, 2.5 times per day instead of 2-3 times per day. For the most extreme category, slightly smaller and larger values were used than the limit for the lowest and highest categories, respectively (e.g., 8 times/week for 7 times/week). Sugar and milk intakes for tea and coffee were calculated from tea and coffee intake. Of the 147 foods in the composition table, the weighted mean was used for 16 which consisted of 2 or more foods (e.g., salted fish, salmon and trout, other oranges) (Table 1). The weighting ratios for the composition of those foods were obtained from the DR data in this validation study, although values rounded off to 60% and 40% for food A and B, respectively, were used because of the limited value of the data.

The significant seasonal variation reported for the intake of some nutrients such as vitamin C, is probably due to the seasonal variation in fruits and vegetables.^3^ For those foods, subjects were asked in the FFQ to report the consumption at the time of year when each food was most available. Seasonal coefficients were used to calculate the average yearly intake of such foods. The coefficients were determined based on the intake reported by the dietary records by season. Area was not considered in this computation. The equation may be stated:

SC_i_ = X_i,ave_ / X_i,max_,

Where SCi is the seasonal coefficient of food_i_, X_i,ave_ is the mean intake of the study subjects for the whole year average estimated from a 28-day (4 season) DR, and X_i,max_ is the mean intake of the population for the intake in a 7-day (1 season) DR of the season with the highest consumption. Table 1 indicates the SCi for each food.

The salt intake from cooking salt and salty seasonings such as soy sauce was estimated from the cooking methods most frequently used for meats, fish, and vegetables, the use of table salt and soy sauce, and the intakes of meats, fish, and vegetables. Intake of cooking oil and salt was estimated by the method used in the dietary history: the amounts of cooking oil and salt for 5 cooking methods (raw, stewed, grilled, deep-fried, stir-fried, and other) were estimated for 3 food groups (meats, fish, and vegetables), and multiplied by the individual intake of each food group according to the cooking methods most frequently used by the individual.^4^ All supplement use and some nutrition-fortified beverages were excluded from the computations in this study because their composition tables were not available.

The intakes of 16 nutrients for each food were calculated using the food composition table developed for the FFQ based on the Standard Tables of Food Composition in Japan, the 4th revised edition.^5^ Since the food composition table for cholesterol has numerous missing values,^6^ we developed a composition table for cholesterol substituting methods used for the development of the fatty acid food composition table.^7^

### Statistical Analysis

The subjects of the present study were 215 persons (102 men and 113 women) with whom both the FFQ for the validation study and their complete dietary records (14-day records in Okinawa and 28-day records in the other 3 areas) were used for the analysis. The mean intakes for food groups and nutrients were calculated by sex and area, and compared by ANOVA. The definition of food groups was mainly based on the Standard Tables of Food Composition in Japan, the 4th revised edition.^5^ Green and yellow vegetables were defined as 44 vegetables with 600 micrograms of carotene per 100 gram and 10 frequently used vegetables that contribute to the intake of carotene among Japanese, according to the definition by the Ministry of Health, Labor and Welfare. Additionally, mugwort (leaves) with 3600 micrograms of carotene per 100-gram food portion was included in the green and yellow vegetables because it could not be overlooked as a carotene source among the subjects in Okinawa in a previous survey.^2^ Salted pickled vegetables were defined according to the food group used in the National Nutrition Survey.^8^ In FFQ, sugar intake was assessed only for those for coffee and/or tea. Therefore, sugar from cooking was not included in the analysis, but the energy and nutrients derived from sugar for coffee and/or tea were included in the corresponding computations.

## RESULTS

Table 1 shows the list of 147 foods used in the computation of intake in the FFQ, their standard portion sizes, and seasonal coefficients. The food codes of the Standard Tables of Food Composition in Japan, the 4th revised edition^5^ used in the computation and their weighting ratios were also described.

Tables 2 and 3 show the intake levels of main nutrients by sex and area. The mean intake of energy was highest in the Ninohe PHC area for men, and in the Saku PHC area for women, and lowest in the Ishikawa PHC area for both men and women. Mean intakes were significantly different among areas for energy and most of the nutrients except alcohol and carotene in both men and women for crude values (p<0.05). After the adjustment for energy intake, the mean intakes were significantly different among areas for protein, total fat, carbohydrate, calcium, phosphorus, sodium, and carotene in both men and women (p<0.05).

**Table 2.** Energy and nutrient intakes assessed with FFQ by area

Sex	Ninohe PHC area				Yokote PHC area				Saku PHC area				Ishikawa PHC area				Total				ANOVA
					
Nutrient	Mean	±	SD	Median	Mean	±	SD	Median	Mean	±	SD	Median	Mean	±	SD	Median	Mean	±	SD	Median	p-value
Men	(n=24)				(n=28)				(n=23)				(n=27)				(n=102)				
Energy (kcal/day)	2744	±	831	2461	2337	±	678	2250	2656	±	503	2632	1760	±	438	1653	2352	±	732	2267	<0.001
Protein (g/day)	112.4	±	50.l	97.7	86.3	±	31.4	77.2	101.7	±	27.3	100.8	62.2	±	22.7	55.1	89.5	±	38.6	79.4	<0.001
Total fat (g/day)	77.7	±	38.5	65.4	57.0	±	25.0	51.4	76.3	±	24.0	73.8	56.3	±	23.6	59.3	66.1	±	29.6	60.6	0.006
Carbohydrate (g/day)	351	±	109	345	323	±	100	305	343	±	66	356	213	±	49	212	305	±	101	290	<0.001
Alcohol (g/day)	23.7	±	26.0	22.7	26.6	±	24.2	23.0	25.7	±	21.1	24.4	19.6	±	22.2	11.7	23.8	±	23.3	22.7	0.705
Calcium (mg/day)	835	±	528	705	632	±	424	536	886	±	278	869	436	±	236	375	685	±	418	594	<0.001
Phosphorus (mg/day)	1765	±	732	1532	1364	±	518	1278	1674	±	387	1638	966	±	335	862	1423	±	595	1329	<0.001
Iron (mg/day)	14.2	±	6.0	13.2	12.4	±	5.9	10.7	13.7	±	4.7	13.1	8.7	±	4.1	7.6	12.2	±	5.6	11.3	0.001
Sodium (mg/day)	6668	±	3091	6020	6008	±	2387	5294	7550	±	3066	7428	3440	±	1512	3261	5831	±	2951	5356	<0.001
Potassium (mg/day)	3634	±	1538	3529	3416	±	1916	3069	3916	±	1305	3821	2394	±	1113	2120	3309	±	1596	3000	0.003
Retinol (*μ*g/day)	951	±	882	692	502	±	415	377	782	±	558	671	435	±	329	364	653	±	602	514	0.005
Carotene (*μ*g/day)	3709	±	2810	3579	3709	±	4603	2341	4939	±	3602	3550	3727	±	2445	3302	3991	±	3477	3315	0.536
Vitamin B_1_ (mg/day)	1.47	±	0.62	1.38	1.24	±	0.54	1.08	1.46	±	0.47	1.36	0.98	±	0.38	0.99	1.27	±	0.54	1.14	0.002
Vitamin B_2_ (mg/day)	2.09	±	0.97	1.97	1.73	±	0.77	1.57	2.09	±	0.66	2.01	1.30	±	0.54	1.21	1.78	±	0.81	1.63	0.001
Niacin (mg/day)	24.7	±	11.6	21.8	20.7	±	7.8	18.9	23.2	±	7.5	23.5	16.2	±	5.7	15.0	21.0	±	8.8	18.7	0.002
Vitamin C (mg/day)	145	±	95	113	197	±	182	169	211	±	108	197	129	±	68	109	170	±	126	156	0.053
Cholesterol (mg/day)	424	±	215	379	295	±	104	289	432	±	199	421	279	±	124	274	352	±	176	327	<0.001

Women	(n=27)				(n=30)				(n=28)				(n=28)				(n=113)				
Energy (kcal/day)	2160	±	691	2107	1974	±	602	1862	2362	±	1297	2073	1584	±	457	1557	2018	±	862	1862	0.005
Protein (g/day)	93.6	±	40.1	85.0	78.3	±	27.4	74.7	102.2	±	73.0	86.5	57.5	±	18.1	55.4	82.7	±	47.1	71.1	0.002
Total fat (g/day)	68.8	±	32.9	56.4	55.8	±	20.8	51.7	80.9	±	58.3	68.1	53.1	±	20.5	52.0	64.5	±	37.5	54.7	0.018
Carbohydrate (g/day)	282	±	72	276	291	±	96	280	307	±	129	268	218	±	58	214	275	±	98	261	0.003
Alcohol (g/day)	4.8	±	14.1	0.0	0.4	±	1.0	0.0	1.0	±	2.4	0.0	0.1	±	0.6	0.0	1.5	±	7.1	0.0	0.053
Calcium (mg/day)	754	±	312	703	627	±	362	555	910	±	589	742	513	±	232	477	699	±	418	596	0.002
Phosphorus (mg/day)	1467	±	537	1404	1241	±	460	1142	1626	±	998	1374	963	±	295	918	1321	±	667	1183	0.001
Iron (mg/day)	13.0	±	5.7	11.0	12.0	±	5.6	10.9	14.8	±	11.1	11.5	8.6	±	2.9	8.5	12.1	±	7.3	10.9	0.010
Sodium (mg/day)	5656	±	2558	4870	5645	±	2267	5060	7327	±	4794	6255	3111	±	1088	2918	5437	±	3308	4730	<0.001
Potassium (mg/day)	3395	±	1441	3077	3298	±	1950	2766	4158	±	2649	3527	2532	±	918	2254	3344	±	1922	2802	0.016
Retinol (*μ*g/day)	805	±	741	576	455	±	431	418	783	±	1036	445	387	±	276	299	603	±	699	427	0.041
Carotene (*μ*g/day)	3653	±	2778	2632	3857	±	4189	2545	4914	±	2815	4269	4248	±	2004	3917	4167	±	3073	3631	0.439
Vitamin B_1_ (mg/day)	1.32	±	0.60	1.12	1.22	±	0.58	1.03	1.51	±	0.87	1.25	0.92	±	0.36	0.82	1.24	±	0.65	1.05	0.006
Vitamin B_2_ (mg/day)	1.76	±	0.72	1.65	1.63	±	0.66	1.52	2.15	±	1.30	1.78	1.35	±	0.48	1.21	1.72	±	0.88	1.52	0.006
Niacin (mg/day)	20.0	±	9.4	17.0	17.2	±	6.5	15.8	23.3	±	17.8	18.9	13.1	±	4.5	12.0	18.3	±	11.3	15.8	0.005
Vitamin C (mg/day)	171	±	105	141	207	±	229	141	255	±	177	197	146	±	91	113	195	±	165	157	0.072
Cholesterol (mg/day)	335	±	186	306	292	±	109	308	418	±	236	388	265	±	130	270	327	±	179	310	0.007

**Table 3.** Energy-adjusted nutrient intakes (energy density) assessed with FFQ by area

Sex	Ninohe PHC area					Yokote PHC area				Saku PHC area				Ishikawa PHC area				Total				ANOVA
					
Nutrient	Mean	±	SD	Median		Mean	±	SD	Median	Mean	±	SD	Median	Mean	±	SD	Median	Mean	±	SD	Median	p-value
Men	(n=24)					(n=28)				(n=23)				(n=27)				(n=102)				
Protein (%E)	15.9	±	3.6	16.1	3.6	14.7	±	2.0	14.4	15.2	±	1.8	15.4	13.9	±	2.5	14.6	14.8	±	2.6	14.8	0.044
Total fat (%E)	24.4	±	6.7	25.0	6.7	21.5	±	4.5	21.9	25.5	±	4.7	25.7	28.0	±	7.7	30.6	24.8	±	6.5	24.7	0.002
Carbohydrate (%E)	51.8	±	7.7	51.8	7.7	55.5	±	7.4	56.1	52.1	±	6.7	53.5	49.1	±	8.4	50.9	52.2	±	7.8	52.4	0.026
Alcohol (%E)	7.1	±	8.0	6.4	8.0	8.1	±	7.4	7.3	6.8	±	5.3	8.0	8.0	±	9.5	4.6	7.5	±	7.7	6.5	0.911
Calcium (mg/1000 kcal)	289	±	111	261	111	257	±	76	251	335	±	88	326	237	±	91	228	277	±	97	261	0.002
Phosphorus (mg/day)	626	±	109	629	109	577	±	64	563	628	±	62	633	540	±	83	539	590	±	88	584	<0.001
Iron (mg/1000 kcal)	5.1	±	1.2	5.4	1.2	5.2	±	1.0	5.1	5.1	±	1.1	5.1	4.8	±	1.3	4.8	5.0	±	1.1	5.0	0.674
Sodium (mg/1000 kcal)	2381	±	641	2325	641	2539	±	485	2500	2791	±	857	2822	1900	±	546	1890	2390	±	707	2359	<0.001
Potassium (mg/1000 kcal)	1290	±	272	1275	272	1410	±	343	1461	1458	±	283	1399	1323	±	343	1321	1369	±	317	1361	0.230
Retinol (*μ*g/1000 kcal)	340	±	262	278	262	215	±	176	142	282	±	167	304	244	±	176	234	267	±	200	232	0.131
Carotene (*μ*g/1000 kcal)	1276	±	634	1296	634	1409	±	1050	1097	1808	±	1074	1365	2049	±	1081	1774	1637	±	1018	1367	0.021
Vitamin B_1_ (mg/1000 kcal)	0.52	±	0.12	0.53	0.12	0.52	±	0.09	0.53	0.54	±	0.09	0.54	0.55	±	0.14	0.55	0.53	±	0.11	0.54	0.779
Vitamin B_2_ (mg/1000 kcal)	0.74	±	0.19	0.72	2.33	0.73	±	0.16	0.72	0.78	±	0.16	0.79	0.72	±	0.16	0.77	0.74	±	0.17	0.75	0.560
Niacin (mg/1000 kcal)	8.8	±	2.3	8.8	27.8	8.8	±	1.7	9.2	8.6	±	1.5	8.6	9.1	±	1.4	8.9	8.8	±	1.7	8.9	0.833
Vitamin C (mg/1000 kcal)	52.5	±	27.8	50.8		79.0	±	39.9	75.1	77.7	±	29.0	70.2	71.2	±	26.8	67.8	70.4	±	32.8	69.2	0.147
Cholesterol (mg/1000 kcal)	149	±	50	160		127	±	36	118	158	±	51	162	155	±	53	150	146	±	49	147	0.085

Women	(n=27)					(n=30)				(n=28)				(n=28)				(n=113)				
Protein (%E)	16.9	±	2.9	16.6		15.8	±	1.8	15.9	16.6	±	2.1	16.8	14.5	±	1.6	14.2	16.0	±	2.3	15.9	<0.001
Total fat (%E)	27.6	±	5.1	27.0		25.4	±	5.4	24.8	29.4	±	6.0	29.2	29.6	±	4.5	29.2	27.9	±	5.5	27.5	0.009
Carbohydrate (%E)	53.6	±	7.0	54.8		58.8	±	6.6	60.1	54.0	±	7.2	54.3	55.5	±	5.5	56.0	55.6	±	6.9	56.5	0.014
Alcohol (%E)	1.6	±	4.8	0.0		0.2	±	0.4	0.0	0.2	±	0.5	0.0	0.0	±	0.2	0.0	0.5	±	2.4	0.0	0.067
Calcium (mg/1000 kcal)	345	±	80	327		309	±	86	285	376	±	92	368	324	±	107	313	338	±	94	321	0.043
Phosphorus (mg/day)	674	±	89	688		626	±	73	624	677	±	67	671	611	±	82	592	646	±	83	646	0.002
Iron (mg/1000 kcal)	5.9	±	1.2	5.8		6.0	±	1.1	5.7	6.0	±	1.3	5.7	5.4	±	0.9	5.2	5.8	±	1.2	5.6	0.206
Sodium (mg/1000 kcal)	2578	±	624	2600		2854	±	642	2768	3021	±	829	2997	1983	±	505	1920	2614	±	762	2597	<0.001
Potassium (mg/1000 kcal)	1541	±	302	1511		1612	±	361	1586	1723	±	311	1719	1598	±	320	1485	1619	±	327	1592	0.215
Retinol (*μ*g/1000 kcal)	358	±	311	255		241	±	246	169	277	±	243	207	246	±	188	185	279	±	251	206	0.285
Carotene (*μ*g/1000 kcal)	1605	±	891	1322		1820	±	1138	1443	2089	±	677	2134	2674	±	1012	2465	2047	±	1018	1901	<0.001
Vitamin B_1_ (mg/1000 kcal)	0.60	±	0.11	0.57		0.60	±	0.12	0.57	0.63	±	0.11	0.60	0.57	±	0.13	0.55	0.60	±	0.12	0.58	0.368
Vitamin B_2_ (mg/1000 kcal)	0.80	±	0.15	0.81		0.82	±	0.18	0.77	0.89	±	0.18	0.87	0.86	±	0.22	0.78	0.84	±	0.18	0.83	0.284
Niacin (mg/1000 kcal)	9.0	±	1.9	8.5		8.7	±	1.3	8.6	9.4	±	1.7	9.3	8.3	±	1.4	8.0	8.8	±	1.6	8.5	0.071
Vitamin C (mg/1000 kcal)	76.2	±	32.3	67.0		95.3	±	52.6	84.1	106.5	±	35.6	101.4	90.5	±	38.6	84.0	92.3	±	41.7	84.1	0.057
Cholesterol (mg/1000 kcal)	149	±	53	147		152	±	56	153	174	±	48	180	168	±	68	171	161	±	57	160	0.306

Tables 4 and 5 show the intake levels of food groups by sex and area. Crude mean intakes were significantly different among areas for food groups except for fats and oils, meats, green and yellow vegetables, algae, and non-alcoholic beverages in both sexes, pulses and alcoholic beverages in men, and milk and dairy products, and vegetables in women (p<0.05). After the adjustment for energy intake, the mean intakes were significantly different among areas for the food groups except for cereals, algae and non-alcoholic beverages in both sexes, pulse, fungi and alcoholic beverages in men, and meat, milk and dairy products and vegetables in women (p<0.05).

**Table 4.** Food group intakes (g/day) assessed with FFQ by area

Sex	Ninohe PHC area				Yokote PHC area				Saku PHC area				Ishikawa PHC area				Total				ANOVA
					
Food group	Mean	±	SD	Median	Mean	±	SD	Median	Mean	±	SD	Median	Mean	±	SD	Median	Mean	±	SD	Median	p-value
Men	(n=24)				(n=28)				(n=23)				(n=27)				(n=102)				
Cereals	404	±	143	380	358	±	110	347	387	±	86	400	258	±	69	247	349	±	118	340	<0.001
Potatoes and starches	22	±	16	19	29	±	27	20	59	±	62	39	21	±	22	19	32	±	38	22	0.001
Sugar and sweeteners		---		---		---		---		---		---		---		---		---		---	---
Confectioneries	11	±	11	11	27	±	37	16	18	±	12	16	9	±	11	8	17	±	22	11	0.013
Fats and oils	14	±	8	12	12	±	7	12	16	±	7	16	14	±	6	13	14	±	7	13	0.211
Nuts and seeds	2	±	2	1	3	±	5	1	4	±	4	2	1	±	1	0	2	±	4	1	0.019
Pulses	41	±	54	27	33	±	28	37	17	±	15	19	21	±	24	14	28	±	34	22	0.064
Fish and shellfish	168	±	125	128	111	±	59	91	127	±	63	114	57	±	31	51	114	±	85	83	<0.001
Meats	76	±	60	57	54	±	35	48	75	±	46	72	81	±	48	73	71	±	48	57	0.177
Eggs	35	±	16	39	25	±	13	25	42	±	28	50	32	±	17	25	33	±	20	25	0.017
Milk and dairy products	253	±	355	128	139	±	148	112	283	±	243	209	124	±	121	72	194	±	237	144	0.033
Vegetables	205	±	114	195	253	±	238	203	345	±	211	294	206	±	136	168	250	±	189	215	0.030
Green and yellow	94	±	66	78	108	±	148	77	151	±	98	128	93	±	66	74	111	±	103	85	0.170
Pickled^1^	27	±	21	19	65	±	66	40	103	±	82	82	6	±	7	5	49	±	64	23	<0.001
Fruits	233	±	244	144	271	±	320	221	227	±	163	178	88	±	73	79	204	±	231	141	0.018
Fungi	10	±	9	9	11	±	8	9	14	±	10	9	7	±	8	3	10	±	9	8	0.043
Algae	11	±	6	11	11	±	7	11	16	±	20	12	9	±	8	6	12	±	12	9	0.249
Alcoholic beverages	293	±	347	180	354	±	332	291	280	±	239	283	297	±	417	90	308	±	339	180	0.867
Non-alcoholic beverages	890	±	729	697	1040	±	757	830	998	±	485	823	707	±	345	617	907	±	611	714	0.191
Seasonings and spices	4	±	4	3	3	±	3	3	7	±	6	6	3	±	3	1	4	±	4	3	0.001

Women	(n=27)				(n=30)				(n=28)				(n=28)				(n=113)				
Cereals	311	±	78	290	307	±	111	281	314	±	106	297	244	±	61	236	294	±	95	276	0.013
Potatoes and starches	32	±	32	23	34	±	32	22	63	±	67	43	22	±	13	22	38	±	43	28	0.002
Sugar and sweeteners		---		---		---		---		---		---		---		---		---		---	---
Confectioneries	15	±	14	12	38	±	37	23	36	±	52	24	18	±	25	11	27	±	36	16	0.028
Fats and oils	15	±	12	11	12	±	7	10	18	±	14	15	13	±	6	12	14	±	10	11	0.085
Nuts and seeds	2	±	2	1	1	±	1	1	7	±	14	2	1	±	1	0	3	±	7	1	0.009
Pulses	36	±	35	27	30	±	26	26	8	±	25	13	17	±	23	11	23	±	29	19	0.001
Fish and shellfish	142	±	114	112	96	±	52	83	141	±	146	112	44	±	23	40	105	±	103	80	0.001
Meats	57	±	43	54	49	±	29	44	72	±	52	56	53	±	34	46	58	±	41	47	0.153
Eggs	25	±	16	25	27	±	16	25	39	±	21	45	33	±	22	32	31	±	19	25	0.027
Milk and dairy products	208	±	130	201	162	±	147	136	270	±	225	209	191	±	193	200	207	±	180	200	0.137
Vegetables	260	±	241	192	259	±	223	228	389	±	306	307	229	±	128	189	284	±	238	233	0.054
Green and yellow	111	±	88	82	114	±	120	83	172	±	122	140	107	±	55	90	126	±	103	103	0.050
Pickled^1^	25	±	19	19	59	±	49	49	98	±	86	74	6	±	6	3	48	±	61	27	<0.001
Fruits	268	±	198	185	319	±	417	215	345	±	330	237	119	±	76	100	264	±	299	178	0.019
Fungi	11	±	9	9	13	±	10	10	17	±	12	16	9	±	7	9	12	±	10	11	0.009
Algae	12	±	7	12	11	±	6	11	15	±	12	12	12	±	10	11	13	±	9	11	0.593
Alcoholic beverages	53	±	132	0	8	±	23	0	16	±	39	0	3	±	16	0	20	±	70	0	0.036
Non-alcoholic beverages	654	±	355	614	874	±	633	667	929	±	580	798	736	±	356	682	801	±	506	689	0.161
Seasonings and spices	4	±	3	4	5	±	4	4	8	±	7	6	3	±	3	3	5	±	5	4	0.002

^1^Pickled plum (umeboshi) was included in pickled vegetables, and not in total vegetables but rather in fruits.

**Table 5.** Food group intakes (g/1000 kcal) assessed with FFQ by area

Sex	Ninohe PHC area				Yokote PHC area				Saku PHC area				Ishikawa PHC area				Total				ANOVA
					
Food group	Mean	±	SD	Median	Mean	±	SD	Median	Mean	±	SD	Median	Mean	±	SD	Median	Mean	±	SD	Median	p-value
Men	(n=24)				(n=28)				(n=23)				(n=27)				(n=102)				
Cereals	151	±	40	156	159	±	54	150	148	±	30	149	151	±	41	148	152	±	42	150	0.780
Potatoes and starches	8	±	6	8	11	±	7	10	22	±	23	18	11	±	8	11	13	±	14	10	0.002
Sugar and sweeteners		---		---		---	---			---		---		---		---	---		---		---
Confectioneries	4	±	5	4	11	±	12	7	6	±	4	6	5	±	5	5	7	±	8	5	0.013
Fats and oils	5	±	2	5	5	±	2	5	6	±	2	6	8	±	3	8	6	±	2	6	<0.001
Nuts and seeds	1	±	1	1	1	±	1	1	1	±	1	1	0	±	1	0	1	±	1	0	0.012
Pulses	13	±	13	9	14	±	12	13	7	±	6	7	12	±	13	9	12	±	12	10	0.118
Fish and shellfish	57	±	33	57	47	±	19	46	46	±	18	45	32	±	16	31	45	±	24	41	0.001
Meats	26	±	16	25	24	±	14	22	27	±	14	28	44	±	21	44	30	±	18	28	<0.001
Eggs	13	±	5	13	11	±	6	10	15	±	8	17	17	±	9	17	14	±	8	13	0.008
Milk and dairy products	86	±	93	62	56	±	42	54	111	±	102	79	67	±	60	42	78	±	78	65	0.059
Vegetables	73	±	33	64	100	±	59	82	128	±	63	112	112	±	53	102	103	±	56	89	0.006
Green and yellow	33	±	20	30	40	±	33	33	55	±	27	47	51	±	27	44	45	±	28	39	0.025
Pickled^1^	10	±	8	8	26	±	23	22	38	±	25	29	3	±	3	3	19	±	22	11	<0.001
Fruits	83	±	76	61	108	±	86	90	82	±	42	72	48	±	37	39	81	±	67	66	0.010
Fungi	4	±	2	3	4	±	3	4	5	±	3	4	4	±	3	2	4	±	3	3	0.186
Algae	4	±	2	4	5	±	3	5	6	±	6	4	5	±	3	4	5	±	4	4	0.639
Alcoholic beverages	117	±	137	77	153	±	138	149	109	±	95	107	172	±	256	58	140	±	170	86	0.517
Non-alcoholic beverages	314	±	198	258	463	±	398	381	377	±	174	318	413	±	208	381	395	±	268	333	0.244
Seasonings and spices	2	±	1	1	1	±	1	1	3	±	2	2	1	±	2	1	2	±	2	1	0.009

Women	(n=27)				(n=30)				(n=28)				(n=28)				(n=113)				
Cereals	150	±	30	146	159	±	43	158	142	±	35	141	159	±	40	159	153	±	38	152	0.270
Potatoes and starches	14	±	10	12	15	±	9	13	26	±	20	21	15	±	8	13	17	±	13	14	0.002
Sugar and sweeteners		---		---		---		---		---		---		---		---		---		---	---
Confectioneries	7	±	5	6	19	±	17	13	13	±	9	9	10	±	10	7	12	±	12	8	0.001
Fats and oils	6	±	3	6	6	±	2	5	7	±	3	7	8	±	2	8	7	±	3	6	0.023
Nuts and seeds	1	±	1	1	1	±	1	1	2	±	3	1	0	±	0	0	1	±	2	1	<0.001
Pulses	18	±	19	15	16	±	13	15	5	±	10	6	10	±	14	8	12	±	15	11	0.004
Fish and shellfish	61	±	37	50	48	±	16	46	54	±	23	50	29	±	12	28	48	±	26	43	<0.001
Meats	25	±	12	23	26	±	15	24	29	±	14	24	32	±	15	31	28	±	14	26	0.246
Eggs	11	±	7	10	14	±	10	13	17	±	7	18	21	±	15	18	16	±	11	14	0.005
Milk and dairy products	99	±	57	91	82	±	59	66	119	±	93	97	123	±	107	126	106	±	82	95	0.212
Vegetables	116	±	82	95	125	±	60	111	158	±	67	145	142	±	54	134	135	±	67	123	0.089
Green and yellow	49	±	32	40	53	±	33	48	71	±	28	69	66	±	24	61	60	±	30	53	0.018
Pickled 1	12	±	9	10	30	±	23	23	41	±	26	34	4	±	4	2	22	±	23	14	0.000
Fruits	118	±	66	116	145	±	107	121	138	±	87	110	73	±	37	57	119	±	83	102	0.004
Fungi	5	±	3	5	6	±	4	5	7	±	3	7	5	±	3	5	6	±	4	5	0.033
Algae	6	±	4	5	6	±	3	5	6	±	3	6	8	±	6	7	6	±	4	5	0.104
Alcoholic beverages	23	±	55	0	5	±	14	0	5	±	10	0	2	±	9	0	8	±	29	0	0.034
Non-alcoholic beverages	313	±	175	279	454	±	378	346	442	±	277	387	506	±	358	419	430	±	314	361	0.132
Seasonings and spices	2	±	1	1	2	±	2	2	3	±	2	2	2	±	1	2	2	±	2	2	0.017

^1^Pickled plum (umeboshi) was included in pickled vegetables, and not in total vegetables but rather in fruits.

## DISCUSSION

Although it was nearly impossible to obtain enough data to develop the FFQ and the attached computation algorithm, a data-based approach was used to the degree possible in developing the questionnaire. First, the food items were listed in order to cover 90% of total intake of energy and 13 nutrients, except for sodium (cover rate was 50%) using the previous data obtained from the subjects in the study areas.^2^ The standard portion sizes, food codes, and their weighting ratios were also basically determined from the data set. The seasonal coefficients were determined from the relative intakes observed in each season in the dietary records of this validation study. We consider this paper to be rather unique in that it presents the questionnaire makeup along with its algorithm for the computation of food intake; nevertheless the validity heavily depends on it. Only food items and the standard portion sizes were described in some reports on the FFQ development (see reference 9).

The most outstanding characteristic of the FFQ may be its use of a seasonal coefficient. Most of the reports from Westem countries concluded that seasonal variation in food and nutrient intake is negligible. On the other hand, some Japanese studies suggested possible seasonal variation in intakes of some foods and, as a result, in some nutrients such as oranges, vitamin C and carotene.^10^ Also in the data of this validation study, a significant seasonal variation was observed for these two nutrients, and their main food sources (i.e., vegetables and fruit).^11^ Therefore, we asked the "frequency of intake for the most available season" for 48 vegetables and fruit. Then we estimated the intakes over the year using the seasonal coefficients: [intake of the most available season]/[intake over the year] obtained from the dietary records in this validation study. This method of using seasonal coefficients to improve the validity of the FFQ was efficient and feasible in our study because the DR data from 4 seasons were available. However, a more precise evaluation of its efficacy is needed to generalize the method. We also estimated intake of cooking oil and cooking salt, and intake of noodle-soup. They were estimated from relatively simple qualitative, rather than quantitative, questions. Because reliability may be compromised by this type of question (see reference 12), further study is needed to assess the possibility and limitations of these questions for inclusion in the computation of intake levels. In estimating the oil and salt used in food preparation, we used answers to questions regarding dietary behaviors, similar to the method employed in the diet history questionnaire.^4^

Cooking oils are major sources of n-6 polyunsaturated fatty acids,^13^ and cooking salts including salty seasonings have been major sources of sodium in a Japanese population.^14^ Therefore, the consideration of these foods seems important when these nutrients are investigated. However, the validity of the method remains to be examined, because the validity for sodium, for example, was relatively low when the 24-hour urinary excretion was used as the gold standard both in the present and previous studies.^15^^,^^16^ The validity and the reproducibility of the FFQ are reported in the other papers in this Supplement.^17^^,^^18^
